# Comparative EEG analysis of Sutra and Tantra meditations: insights into meditative states and long-term traits

**DOI:** 10.3389/fpsyg.2026.1846053

**Published:** 2026-10-28

**Authors:** Julia Boytsova, Ngawang Norbu, Lodoe Sangpo, Elena Kokurina, Chhoktan Lama, Tanzin Chhonden, Yeshi Dorjee, Yeshi Dorjee, Jamyang Jamyang, Lobsang Jangchup, Lobsang Jinpa, Jampa Khechok, Stanzin Lhakpa, Lharkyab Lharkyab, Kunga Lhundup, Tenzin Lobsang, Tanzin Mutup, Thupten Namdol, Rigzin Nurbu, Lobsang Phuntsok, Thupten Sherap, Jampa Soepa, Jampa Thakchoe, Tenzin Wangdu, Valery Ponomarev, Yu D. Kropotov, Svyatoslav Medvedev

**Affiliations:** 1 Department of Psychology and Psychophysiology, Institute of Biomedical Problems of the Russian Academy of Sciences, Moscow, Russia; 2 Sera Je Monastic University for Advanced Buddhist Studies and Practice, Bylakuppe, Mysore, India; 3 Gaden Jangtse Monastic College, Mundgod, India; 4 Academician Natalya Bekhtereva Foundation, Saint Petersburg, Russia; 5 Gaden Shartse Monastic College, Mundgod, India; 6 Drepung Gomang Monastic College, Mundgod, India; 7 Gyuto Tantric Monastery, Sidhbari, India; 8 Drepung Loseling Monastic University, Mundgod, India; 9 Tashi Lhnpo Monastic University, Bylakuppe, India; 10 Sera Mey Monastic University, Bylakuppe, India; 11 Gyudmed Tantric University, Hunsur, India; 12 N.P. Bekhtereva Institute of the Human Brain of the Russian Academy of Sciences, Saint Petersburg, Russia

**Keywords:** EEG spectral power, individual Alpha Peak Frequency (iAPF), meditative states and traits, Tibetan Buddhism, Sutra and Tantra

## Abstract

**Introduction:**

The Tibetan Buddhist tradition distinguishes between two broad categories of meditation: Sutra and Tantra, which differ in the methods and mental faculties they employ. The main neurophysiological studies of Tibetan Buddhist meditation have been conducted mostly on Sutra practices; while Tantric meditation remains less studied.

**Methods:**

This publication presents a dual comparative study, using both between-groups and within-subjects design, of the neurophysiological (EEG) correlates characterizing Sutra and Tantra meditative practices, examining both the meditative states and the meditative traits. The study was carried out within the natural environment of Tibetan Buddhist monasteries in India, maintaining the socio-cultural context. Two groups of long-term practitioners (Buddhist monks of Gelug lineage), one experienced in the Tantra tradition (T-group, *n* = 21) and the other in the Sutra tradition (S-group, *n* = 20) participated in the study.

**Results:**

A between-group cluster permutation test revealed that the groups differed in EEG alpha power during meditation, as well as in baseline resting conditions before and after meditation. Specifically, T-group demonstrated consistently lower alpha power compared to the S-group, regardless of the conditions. Thus, the observed differences are stable characteristics of the groups. The within-subjects analysis demonstrated that the conditions (meditation, and baseline resting states before and after meditation) differed only in the T-group: during meditation, an increase in individual alpha peak frequency (iAPF) was observed in the occipital leads, followed by a decrease in iAPF after meditation.

**Discussion:**

We suggest that reported between-group differences represent enduring meditative traits developed through years of training in meditative techniques in the Sutra and Tantra traditions. Nevertheless, we cannot entirely exclude the possibility that these variations may also stem from certain cohort-specific characteristics unrelated to meditation. An examination of individual power spectra revealed that the absence of significant differences in EEG spectral power between conditions can be primarily attributed to the inter-individual variability in both groups. This variability, in turn, may stem from extensive expertise of highly experienced practitioners, who over years of training, develop individualized cognitive strategies for maintaining the meditative state. Our future research will focus on a more detailed quantitative analysis of inter-individual variability during meditations and on identifying additional between-group differences among practitioners in different traditions.

## 1 Introduction

Neurophysiological studies of meditation and its effects on the human state are extensively represented in the literature. However, the heterogeneity of meditative practices, their cultural context, and the lack of clear classification limit the possibility to analyze and generalize findings, as well as to evaluate their long-term effects. In this regard, comparative studies of the neurophysiological correlates of various meditation types, conducted within a uniform socio-cultural context and a standardized format, both during the meditative state itself and during pre-meditation and post-meditation resting baseline sessions, are of particular relevance.

For instance, the Mahayana Tibetan Buddhist tradition distinguishes between two primary categories: Sutra and Tantra. Consequently, there are two main groups or classes of meditative practices belonging to the categories of Sutra and Tantra, respectively (Donets, 2007).

The majority of neurophysiological studies on Tibetan Buddhist meditations have been focused specifically on Sutra practices. Within this class, two main groups can be distinguished: Focused Attention Meditation (FAM) (Lutz et al., 2008) and analytical meditation.

Focused Attention Meditation entails voluntary focusing of attention on a chosen object in a sustained manner. The meditator initially focuses attention on the breath or another simple object, and each time the mind becomes distracted and begins to wander from one thought to another, the meditator should become aware of this, detach from the content of the distraction, and refocus attention on the object of meditation (Wallace, 2009; Dalai Lama et al., 2019). With experience, distractions become less frequent and the mind can achieve deeper states of focused absorption, characterized by mental stability, which allows one to concentrate on more complex, abstract internal objects, such as the Buddhist philosophical concepts of emptiness (Sanskrit: *shunyata*) or impermanence (Sanskrit: *anitya*) (Wallace, 2009; Dalai Lama et al., 2019). EEG studies demonstrate that this meditation correlates with increased alpha and theta activity compared to the resting state (Lomas et al., 2015). It is also associated with enhanced gamma activity in the posterior cortex (Lee et al., 2018). Such simultaneous presence of gamma, alpha, and theta activity may indicate a state of “heightened awareness, relaxed alertness, and well-being” (Lomas et al., 2015).

The so-called analytical meditation (Sanskrit: *vipashyana*, not to be confused with *vipassana* in the Theravada tradition) (Wallace, 2009; Dalai Lama et al., 2019; Pabongka, 2006) is a mental training method that combines deep concentration with active reflection. In this practice, the mind employs analysis to understand the nature of the mind and reality. In Tibetan Buddhism, it is used to deconstruct philosophical ideas or concepts into their constituent parts, analyze them, and gain insight into their essence. Analytical meditation can be practiced either independently or as a preparatory stage preceding FAM on the same internal object (compassion, impermanence, emptiness), and as a tool for restoring focus if the practitioner becomes distracted from the object during one-pointed meditation (Dalai Lama et al., 2019). Studies of analytical meditation are less frequently represented in neurophysiological research compared to FAM. One example includes relatively recent works investigating a dynamic variant of analytical meditation (Gelug tradition) during monastic debates using EEG hyperscanning methods (van Vugt et al., 2020). In another study (Neri et al., 2024) comparing the neurophysiological correlates of analytical meditation and FAM, analytical meditation–unlike FAM–did not exhibit pronounced changes in EEG power parameters when compared to the resting state. As for meditative practices within the Tantric class, particularly those belonging to Anuttarayoga Tantra (Tsongkhapa, 2016), they still maintain their sacred status. These practices are subject to direct transmission from teacher to student and, as a result, remain the least integrated into neurophysiological research. A distinctive feature of Tantric meditative practices, compared to those of the Sutra class, is the inclusion of a broader range of qualitatively different “non-verbal” tools, such as symbols, emotions, and more complex visualizations, including somatic (body-oriented) ones. At the same time, Tantra meditations are to some extent based on skills developed through Sutra practices - the development of sustained concentration and the ability to use analytical techniques to deeply penetrate the core concepts of Buddhist philosophy. Specific techniques of Tantric practice generally include Deity Yoga and related complex visualization techniques (Donets, 2007; Tsongkhapa et al., 2013; Blo-Bzacn-Byams and Engle, 2019). Most of studies of meditative practices within the Tantric tradition focus on the primary tool of Tantra - tantric visualizations. Thus, during tantric visualizations of deities at the “self-generation” and “self-dissolution” stages, activation of the right frontotemporal cortex, which is involved in self-perception and the perception of surrounding reality, was observed (Lehmann et al., 2001). In another study (DeLuca John and Daly, 2023), EEG parameters were compared in a single highly experienced practitioner during two distinct types of tantric visualization: (1) visualizing the image of a Deity-Yidam in front of oneself; and (2) visualizing oneself manifesting as the Deity-Yidam (“self-generation” of the Deity). The second type, in contrast to the first, was accompanied by a decrease in central delta and posterior alpha amplitudes, as well as an increase in beta amplitude in the central region of the right hemisphere. It is also worth mentioning recent studies by members of our research group (Mikhaylets et al., 2024, 2026), which examined the sequential structure of the stages of the same tantric practices as in the present study, based on EEG features.

Some previous studies have indicated neurophysiological differences between meditative practices in the Sutra and Tantra classes. A comparative study (Amihai and Kozhevnikov, 2014) demonstrated that tantric meditations trigger an arousal response, in contrast to Sutra-class meditations. In another study (Kozhevnikov et al., 2022), it was found that Tantric practices induced a phasic alertness and an increase in alpha2 power. In contrast, Sutra meditation was characterized by tonic alertness, and active monitoring, as suggested by an increase in alpha1 power.

Measuring brain responses to meditative practice is based on the premise that different conscious states during meditation are accompanied by different neurophysiological states and on reports that meditation practice induces distinct states and traits of consciousness. State refers to the altered sensory, cognitive, and self-referential awareness that can arise during meditation, whereas trait denotes the lasting changes in these dimensions that persist irrespective of active practice (Cahn and Polich, 2006). Regular meditation practice can elicit both transient short-term states enduring, as long-term traits (Cahn and Polich, 2006; Thomas and Cohen, 2014).

Neurophysiological studies of meditative states are typically conducted using a within-subject design, comparing the meditative state to a baseline control state outside of meditation (Travis et al., 2010; Lutz et al., 2004; Huang and Lo, 2009; Sharma et al., 2018; Kakumanu et al., 2018; Aftanas and Golocheikine, 2001, etc.). In contrast, studies of meditative traits are carried out through several approaches: for instance, via between-groups design comparing practitioners and non-practitioners in a baseline control state (Tebecis, 1975; Orme-Johnson and Haynes, 1981; Pagano and Warrenburg, 1983; Travis, 1991; Khare and Nigam, 2000; Lutz et al., 2004; Aftanas and Golocheikine, 2005; Tei et al., 2009; Huang and Lo, 2009; Berkovich-Ohana et al., 2013; Sharma et al., 2018, etc.). Furthermore, some researchers compare groups of more and less experienced meditators across two conditions–during meditation and at rest (e.g., Corby et al., 1978; Aftanas and Golocheikine, 2001; Irrmischer et al., 2018). Finally, longitudinal studies examine the same group before and after a meditation training course (Satyanarayana et al., 1992; Deepak et al., 1994; Davidson et al., 2003; Tomljenović et al., 2016; Rusinova et al., 2024, etc.). In these cases, between-group neurophysiological differences are interpreted as long-term changes in brain activity resulting from increased meditation experience.

It is important to note that most studies focusing on Tantric meditative techniques investigate the neural correlates of meditative states - conscious experiences occurring specifically during the practice. In contrast, research on various types of Sutra-based meditation (especially mindfulness in the Western cultural context) addresses both the immediate effects of meditation and the long-term changes resulting from sustained practice, which are linked to neuroplasticity (meditative traits) (Cahn and Polich, 2006; Thomas and Cohen, 2014; Atad et al., 2025). Consequently, the long-term neurophysiological impact of Tantric practices remains significantly less understood.

It has been shown that meditative states and traits are primarily manifested in an increase in EEG alpha power (Travis, 1991; Satyanarayana et al., 1992; Deepak et al., 1994; Khare and Nigam, 2000, Cahn and Polich, 2006; Fell et al., 2010). Furthermore, some EEG studies indicate that both short-term and long-term meditative changes are associated with increased theta power (Tebecis, 1975; Pagano and Warrenburg, 1983; Aftanas and Golocheikine, 2001, 2005; Sharma et al., 2018; Rusinova et al., 2024), although divergent findings also report a decrease in theta activity (Tomljenović et al., 2016). Changes in beta activity have also been identified as a signature of meditative traits, with reports showing both increases (Huang and Lo, 2009; Lavallee et al., 2011; Thomas et al., 2014) and decreases (Pasquini et al., 2015) in beta power. Therefore, in this study, we also aimed to identify differences in EEG parameters across both groups and conditions.

In the present work, we conducted a dual comparative study, using both between-groups and within-subjects design. We hypothesized that comparing EEG activity before, during, and after meditation across conditions within each group would allow us to identify meditative states specific to each meditation tradition. We hypothesized that between-groups neurophysiological differences would elucidate the long-term changes in brain function arising from long-term experience in the different traditions. We compared two groups of experienced practitioners - a Sutra group (S-group) and a Tantra group (T-group). Given that Tantric meditations rely inherently on foundational skills developed through Sutra practice, the Sutra group serve as a control for the Tantra practitioners.

Given that a number of controversial and open questions still persist in meditation research, we considered it essential to conduct our study within the framework of a uniform socio-cultural context familiar to the subjects - specifically, within monasteries that serve as guardians of the tradition and ensure a uniform format and quality of meditative practices. On one hand, these include the major monastic universities of the Gelug tradition (Sera Je, Sera Mey, Tashi Lhunpo, Drepung Gomang, Drepung Loseling, Gaden Jangtse, and Gaden Shartse), where Focused Attention (FAM) and analytical meditations are taught and practiced. On the other hand, we included the Tantric colleges of the same tradition (Gyuto and Gyudmed), where the core practices center on the three main deities: Guhyasamāja, Yamāntaka, and Chakrasamvara Tantras (Blo-Bzacn-Byams and Engle, 2019; Tsongkhapa et al., 2013). Thus, the studied groups of practitioners are comparable in such parameters as education, lifestyle, and religious beliefs.

The study involved experienced monks from these monasteries, performing meditative practices in the Sutra and Tantra categories, respectively. For the first group, the core practices consist of analytical meditation and/or FAM, while for the second, it is tantric practice on one of the three main deities (Guhyasamāja, Yamāntaka, or Chakrasamvara Tantra). It should be noted that the research team included specially trained monastic researchers representing the main monasteries of the Gelug tradition.

## 2 Materials and methods

### 2.1 Meditations

Practices belonging to the Sutra tradition include one-pointed concentration [classified as FAM by Lutz et al. (2008)] and analytical meditation. During FAM, the practitioner focuses and sustains attention on a single internal object. In the context of the regular practice observed in our participants, this concentration was directed toward key Buddhist concepts, such as compassion (karun_._ā), emptiness (śūnyatā), or impermanence (anitya) (Wallace, 2009; Dalai Lama et al., 2019). Analytical meditation is generally an analytical process that leads to a deep understanding of the basic philosophical concepts of Buddhism. Analytical meditation can be practiced as a preliminary stage to FAM. The analytical process can also be used to restore focus if the meditator has become distracted from the object of concentration during FAM (Wallace, 2009; Dalai Lama et al., 2019; Pabongka, 2006).

Although some neurophysiological differences between analytical meditation and one-pointed concentration have been previously identified (Neri et al., 2024), we did not differentiate between these practices in the current study. Participants were instructed to perform their customary daily meditation as per their usual routine. Post-session interviews revealed that many practitioners periodically integrated analytical meditation during sessions of FAM to refine or restore their focus of concentration. Therefore, at this stage of the study analytical meditation and FAM were not separated but were combined under the broader category of Sutra-based practices.

Practices belonging to the Tantra tradition (the practices of the three main deities: Guhyasamāja, Chakrasamvara, and Yamāntaka/Vajrabhairava) follow a rigorous protocol established in the Buddhist tradition and practiced over centuries (Tsongkhapa et al., 2013; Blo-Bzacn-Byams and Engle, 2019). These practices differ in the iconographic form of the Deity, the granularity of individual mental imagery, and specific methodological emphases. However, they are all classified within the same system of Anuttarayoga Tantra and all include the core part of practice - “taking the Dharmakāya of death into the path (Tibetan: *’chi ba chos sku’i lam ‘khyer*) which unfolds through eight sequential stages of the “dissolution of bodily elements and mental states,” culminating in the state of “Clear Light.” All these practices involve a complex imaginative process of “self-generation,” which entails traversing the eight stages of dissolution in reverse order (Tsongkhapa et al., 2013). These Tantric meditations also rely on the foundation of FAM, as sustained attention is essential for maintaining complex mental imagery during the practice (e.g., the visualization of a Deity surrounded by its retinue). However, in this context, the visualized images do not remain static (as in traditional one-pointed concentration meditation); instead, they undergo specific transformations in accordance with the liturgical text of the practice (Sādhanā). Since Tantric practices are performed based on a scriptural text held in the practitioner’s memory, this practice encompasses not only visualization but also processes of sequential retrieval of information from long-term memory. Furthermore, at specific stages of Tantric meditations, the practitioner may generate and experience distinct emotional states while visualizing various processes within the physical body related to “subtle body” anatomy. Consequently, beyond simple concentration, the investigated Tantric practices encompass a diverse range of cognitive processes and involve complex sequential stages (Tsongkhapa et al., 2013; Blo-Bzacn-Byams and Engle, 2019; Kittay and Lobsang, 2020).

### 2.2 Participants

Forty-one experienced monks-practitioners (all male, ethnic Tibetans) participated in the study. Data collection was conducted over a 3-years period during several field expeditions to Tibetan Buddhist monasteries of the Gelug lineage (Sera Je, Sera Mey, Gaden Jangtse, Gaden Shartse, Drepung Gomang, Drepung Loseling, Tashi Lhunpo, Gyuto, and Gyudmed) in Southern and Northern India. Research was carried out within the authentic cultural environment and conditions familiar to the subjects’ daily meditative practice. Details on the subject’s population are reported in Table 1.

**TABLE 1:** Study population.

ID (T-group)	Age (years)	Regular practice (years)	Meditation duration (sec)	Medicines	Meditation type	Meditation success	Discomfort	Drowsiness, fatigue	Time	Retreat periods
1	46	22	1744	No	1	3	2	1	2:30 PM	2, 5 months
2	52	30	1390	Tibetan medicine	2	1	2	1	2:30 PM	2 years
3	43	4	1786	No	2	1	1	1	2:30 PM	3 years
4	54	10	1036	No	1	2	1	1	8:30 AM	3 years
5	62	23	1294	No	2	1	2	1	2:30 PM	3 years
6	55	21	2024	No	2	3	1	1	10:00 AM	2–3 months
7	57	9	752	Western medicine (BP pills)	2	2	1	1	8:30 AM	2–3 months
8	59	15	1169	Tibetan medicine	2	3	1	1	8:30 AM	2–3 months
9	50	15	2268	No	2	N/A	N/A	N/A	8:30 AM	2–3 months
10	50	21	3916	No	1	3	2	1	8:30 AM	2–3 months
11	54	10	663	No	1	2	2	1	10:00 AM	3 years
12	37	14	786	No	1	1	2	1	8:30 AM	6 months
13	52	26	781	No	1	1	2	1	8:30 AM	4 months
14	41	11	1333	No	1	1	2	1	8:30 AM	No
15	53	21	923	No	2	2	1	1	8:30 AM	2–3 months
16	50	9	3117	Tibetan medicine	2	2	1	1	8:30 AM	3 years
17	58	8	467	No	1	N/A	N/A	N/A	3:00 PM	6 years
18	58	8	2231	No	1	1	1	2	8:30 AM	6 years
19	47	16	1768	No	1	3	2	1	8:30 AM	2–3 months
20	58	32	2571	Western medicine (sugar level)	2	2	1	N/A	2:30 PM	6 years
21	60	12	3331	Tibetan medicine	2	1	N/A	N/A	2:30 PM	3 years
ID (S-group)	Age	Regular practice (years)	Meditation duration (sec)	Medicines	Meditation type	Meditation success	Discomfort	Drowsiness, fatigue	Time	Retreat periods
1	59	22	557	No	1	2	1	1	2:30 PM	8 years
2	49	3	1344	No	3	3	1	1	9:30 AM	No
3	55	5	1731	Western medicine (BP pills)	2	2	2	1	3:00 PM	4 years
4	45	2	2212	No	3	1	2	1	9:00 AM	1 year
5	54	2	2084	No	1	1	2	2	9:00 AM	N/A
6	49	11	1817	No	2	3	2	1	10:00 AM	No
7	45	15	1763	No	2	3	2	N/A	2:30 PM	10 days
8	43	10	3770	No	2	3	2	1	8:30 AM	8 years
9	49	2	2620	No	3	2	2	1	8:30 AM	18 days
10	42	20	956	No	2	2	1	2	2:30 PM	No
11	45	10	1735	No	1	1	2	2	2:30 PM	1 month
12	53	4	912	Tibetan medicine	2	2	2	2	9:00 AM	20 days
13	57	13	1557	Tibetan medicine	2	2	1	1	8:00 AM	No
14	60	24	935	No	2	3	2	1	8:30 AM	2 weeks
15	30	10	1251	No	2	1	2	N/A	9:30 AM	No
16	57	6	1177	Western medicine (sugar level)	3	2	1	1	8:30 AM	2 months
17	50	25	1144	Tibetan medicine, western medicine (BP pills)	2	1	2	N/A	2:30 PM	2–3 months
18	55	12	2216	Western medicine (against cold)	2	2	2	N/A	5:00 PM	2–3 months
19	50	16	2935	No	2	3	2	2	8:30 AM	2 months
20	44	6	759	No	1	1	1	N/A	8:30 AM	2 years

For each subject ID, age (years), duration of meditation session (sec.) are reported. Regular practice describes the number of years of regular practice. In medicine, the presence/absence of drugs taken on a regular basis or on the day of the study is described. Meditation type describes the meditation performed during investigation; in T-group it can be 1 – Guhyasamāja and 2 – Yamāntaka, in S-group it can be 1 - one-pointed concentration, 2 - analytical meditation, or 3 - mixed meditation, when practitioners periodically switched to analytical meditation during one-pointed concentration to restore their focus. Meditation success describes the subjective level of success of a meditation practice and can be among 3 – better than usual, 2 – as usual, 1 – worse than usual. Discomfort describes the 1 - absence / 2 - presence of subjective estimation of anxiety from the EEG recording procedure or discomfort from the EEG cap, as well as subjective estimation of physical discomfort (back pain, knee pain, stiff body, etc.). Drowsiness/fatigue describes the 1 - absence / 2 - presence of a subjective feelings at the time of the study. Time indicates the start time of the study. N/A, not applicable.

Participants were divided into two groups. T-group consisted of 21 monks who performed tantric practices: 10 subjects performed the Guhyasamāja practice, 11 subjects performed the Yamāntaka practice. All participants in this group identified these tantric meditations as their core daily practice. It is noteworthy that almost half of the monks in the T-group had completed a traditional long-term 3-years retreat focused on these specific tantric systems. S-group consisted of 20 monks. During the study, 4 participants performed FAM, 12 - performed analytical meditations and 4 subjects performed a mixed type of meditation, when during FAM the subject periodically switches to the analysis process to restore the focus of concentration. It is important to note that while some participants in this group had obtained the Geshe Lharampa degree and completed the mandatory 1-year tantric course at a tantric college, they had not engaged in formal tantric practice for more than 10 years prior to the study. Furthermore, they identified non-tantric (Sutra-based) meditations as their primary and consistent daily practice.

Prior to the study, all participants were informed of its objectives and procedures and provided written informed consent. The study protocol was designed and conducted in accordance with the ethical standards of the Declaration of Helsinki (1964) and its later amendments. The research was approved by the Ethics Committee of the N.P. Bekhtereva Institute of the Human Brain of the Russian Academy of Sciences (RAS). The informed consent form was originally written in English. Since some participants were not proficient in English, the document was translated and explained in detail in their native Tibetan language. This process was facilitated by monk-researchers fluent in both English and Tibetan, ensuring that all participants fully understood the nature of the study before signing.

### 2.3 Procedure

Before and after each research session, participants took part in an interview based on a structured, unified questionnaire. These interviews were conducted in the Tibetan language by the monks-researchers with the assistance of a translator. The results of the structured interview are presented in Table 1.

The first part of the survey collected biographical data and detailed information regarding the duration and specific content of the subjects’ regular meditative practice. This was aimed at assessing the practitioners’ individual expertise and confirming their categorization into either the Sutra or Tantra group. Additionally, the initial assessment included screening for chronic and neurological conditions to ensure the participants met the study’s inclusion criteria.

The study was conducted in a comfortable environment for the meditators; the practitioners sat in their usual meditation pose – on a mat with legs crossed (lotus position). Subsequently, participants performed their customary meditation practice with eyes closed. There were no time limits imposed on the meditation session; however, a specific constraint was required: subjects performed a 10-min baseline resting-state recording (with eyes open – 5 min and eyes closed – 5 min) immediately before and after the session (pre-meditative and post-meditative control states). The duration of meditation sessions varied from 20 to 60 min determined by each participant’s individual practice pacing.

It should be noted that in this study, we compared the two groups across three conditions: pre-meditation, meditation, and post-meditation states – all with eyes closed. Consequently, participants who practiced meditation with their eyes open were excluded from the analysis to ensure physiological consistency. We also analyzed EEG parameters during meditation relative to the baseline resting states (quiet wakefulness with eyes closed) both before and after the session within each practitioner group.

Following the session, each practitioner participated in a debriefing aimed at re-exploring their contemplative experience and the specific experiential events encountered during the meditation state.

### 2.4 EEG recording

EEG was recorded from 19 scalp wet electrodes positioned according to the International 10–20 system (Fp1, Fp2, F7, F3, Fz, F4, F8, T3, C3, Cz, C4, T4, T5, P3, Pz, P4, T6, O1, O2). Data were acquired using a Mitsar-EEG-201 computer electroencephalograph (Mitsar Co., Ltd., Saint Petersburg, Russia) and an MCScap electrode cap. The signal was sampled at 500 Hz and referenced to linked earlobes (monopolar montage), with the ground electrode located at the FPz position. A digital notch filter was applied to the data at 45–55 Hz to remove artifacts from nearby electrical devices. The resistance of the electrodes did not exceed 10 kOhm.

### 2.5 EEG pre-processing

EEG data pre-processing was carried out using the WinEEG software package (developed by V. A. Ponomarev & J. D. Kropotov, RF2001610516, 2001). To ensure spatial neutrality of the reference and minimize local artifact influence, the raw EEG data were converted to a weighted average montage (Av). During EEG signal processing the high-pass filter (1.5 Hz) and the low-pass filter (40 Hz) was applied. The independent component analysis (ICA) was employed to automatically correct artifacts associated with eye movements, blinks, cardiac activity and muscle contractions using the WinEEG software package. The Infomax algorithm was employed for the ICA component decomposition. After automated correction, all EEG recordings were subjected to rigorous visual inspection to identify any remaining motion artifacts. EEG segments containing such artifacts were manually excluded from subsequent analysis.

Since the subjects were performing their habitual meditation practice without predefined temporal limitations, the duration of meditation varied from 20 to 60 min across subjects. Consequently, while the duration of meditation was not standardized this approach maximized the ecological validity of the study. To achieve standardization, we implemented an automated procedure to adjust the number of artifact-free epochs in the meditation condition. For this purpose, we initially quantified the number of artifact-free epochs for each subject during the meditation condition. The average yield across all participants was 600 epochs. Therefore, the spectral analysis of the EEG during meditation was limited to 600 epochs. The final number of artifact-free epochs included in the meditation analysis was 413 for the T-group and 527 for the S-group. In the resting states before and after meditation, the final number of epochs in the T group were 130 and 134, respectively, while in the S group, they were 135 and 131.

For each condition (before, after, and during meditation) and each subject, individual EEG power spectra were calculated using the following parameters: epoch length of 4 s, overlap of 50%, and Hann window (WinEEG software package was used). The individual power spectra were then averaged for each group of subjects in each condition.

### 2.6 EEG analysis

To identify differences in EEG absolute spectral power both between and within the participants’ groups, a cluster-based permutation test (implemented in the WinEEG software package) was employed. Cluster-based permutation analysis (Maris and Oostenveld, 2007) was carried out for the EEG power spectra within 1–30 Hz frequency band. The clustering parameters were defined as follows: the cluster-forming threshold was set to an 0.05, with a minimal acceptable cluster size – 30 and a spatial extent of at least two adjacent EEG channels, a number of balanced permutations - 10 000, acceptable statistical significance threshold for clusters comparison *p* < 0.05, statistics for between group comparison – Mann Whitney test, for within-group comparison – Wilcoxon signed test. For the differences identified between clusters, in addition to the *p*-level, the values of the effect size are also indicated - Partial eta squared (η2ρ).

Since examination of individual power spectra in the pre-meditation, meditation, and post-meditation conditions revealed a marked variation in the frequency of the maximum spectral peak of the alpha rhythm (8–13 Hz) in the occipital EEG leads (O1 and O2), further analysis of the peak frequency values of the alpha rhythm (individual Alpha Peak Frequency - iAPF) between conditions and between groups was conducted. The individual alpha peak frequency values were calculated using data from the occipital electrodes (O1 and O2), where the alpha power peak was observed most consistency across all subjects and all conditions. The obtained values were then averaged for the O1 and O2 leads. These individual values of iAPF were subjected to nonparametric statistical analysis (Friedman ANOVA) to identify differences between the three conditions (pre-meditation, meditation, and post-meditation) separately within each group of subjects. To perform within-group *post hoc* comparisons, the paired Wilcoxon signed-rank test was utilized. Inter-group differences in the individual alpha peak frequency were identified using the paired nonparametric Mann-Whitney U Test. The results also include effect size values (Cohen’s d). To account for the multiple comparisons problem, the significance level (*p*-level) was adjusted using the Bonferroni correction, where the initial *p*-value was multiplied by the number of comparisons performed.

## 3 Results

### 3.1 Between-group cluster-based permutation analysis

Table 2 presents the alpha rhythm (8–13 Hz) power values in each condition for each group of subjects.

**TABLE 2:** Alpha rhythm power values (8–13 Hz) in the occipital EEG electrodes (O1 and O2) in each condition for each group of subjects.

	Group S (*n* = 20)		Group T (*n* = 21)	
	O1 (uV^∧^2)	O2 (uV^∧^2)	O1 (uV^∧^2)	O2 (uV^∧^2)
Pre-meditation	26.9 ± 20	25.6 ± 19	11.9 ± 9.7	11.6 ± 9.1
Meditation	30.8 ± 25	27 ± 20	13.3 ± 9.5	13.7 ± 10.2
Post-meditation	31.5 ± 25	28.4 ± 21	15.5 ± 13.4	15.2 ± 13.5

Mean values and standard deviations are presented.

The between-group cluster-based permutation analysis revealed significant differences in alpha power across all three conditions.

#### 3.1.1 Pre-meditation resting baseline

A significant cluster was detected in the 9.3–11.5 Hz frequency band (cluster size = 130, *p* = 0.014, η^2^_ρ_ = 0.22). The T-group exhibited significantly lower alpha power compared to the S-group; these differences were distributed across most cortical areas (Figure 1A).

**FIGURE 1:**
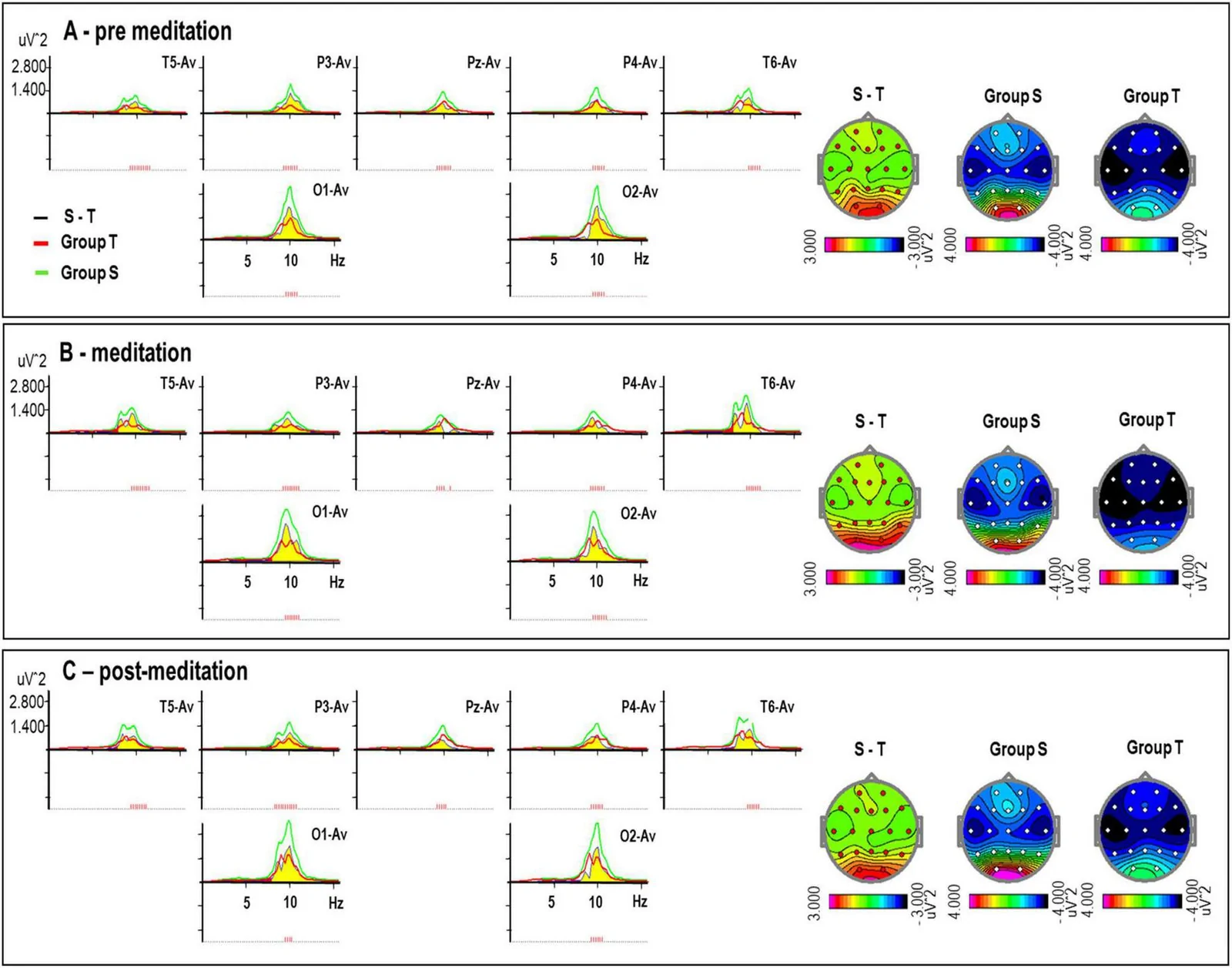
The between-group cluster permutation analysis in EEG spectral power **(A)** in the pre-meditation resting baseline session, **(B)** during meditation, **(C)** in the post-meditation resting baseline session. The graphs of the EEG spectral power are presented (red – group T, green - group S, black – differences). Scalp topographies show the statistic comparing group S vs. group T, red electrodes show presence of an observed effect in between-group cluster-permutation analysis. The vertical red lines on the *x*-axis indicate the cluster boundaries (*p* ≤ 0.05).

#### 3.1.2 Meditation

During the meditation session, a significant cluster was identified in the 9.3–11.5 Hz frequency band (cluster size = 140, *p* = 0.017, η^2^_ρ_ = 0.20). In the T-group, lower alpha rhythm power was again observed in most cortical areas (Figure 1B).

#### 3.1.3 Post-meditation resting baseline

Comparison of the two groups following meditation also revealed a significant cluster in the 8.3–11.2 Hz frequency band (cluster size = 118, *p* = 0.018, η^2^_ρ_ = 0.16). Consistent with the previous conditions, T-group maintained lower alpha rhythm power values (Figure 1C).

### 3.2 Within-group cluster-based permutation analysis

Examination of individual EEG power spectra in the S-group and T-group revealed some differences between conditions (pre-meditation baseline, meditation, and post-meditation baseline) at the subject level. For instance, Figures 2, 3 displays the individual EEG power spectra for each practitioner from both groups at electrodes T5, P3, Pz, P4, T6, O1 and O2, illustrating variability of individual EEG power spectra. In contrast, the group-averaged EEG power spectra for each of the three conditions, shown in Figure 4 demonstrates no significant differences between conditions. Furthermore, within-group cluster-based permutation analysis also failed to identify any significant changes in EEG power when comparing the three conditions (pre-meditation baseline, meditation, and post-meditation baseline) within each practitioner group.

**FIGURE 2:**
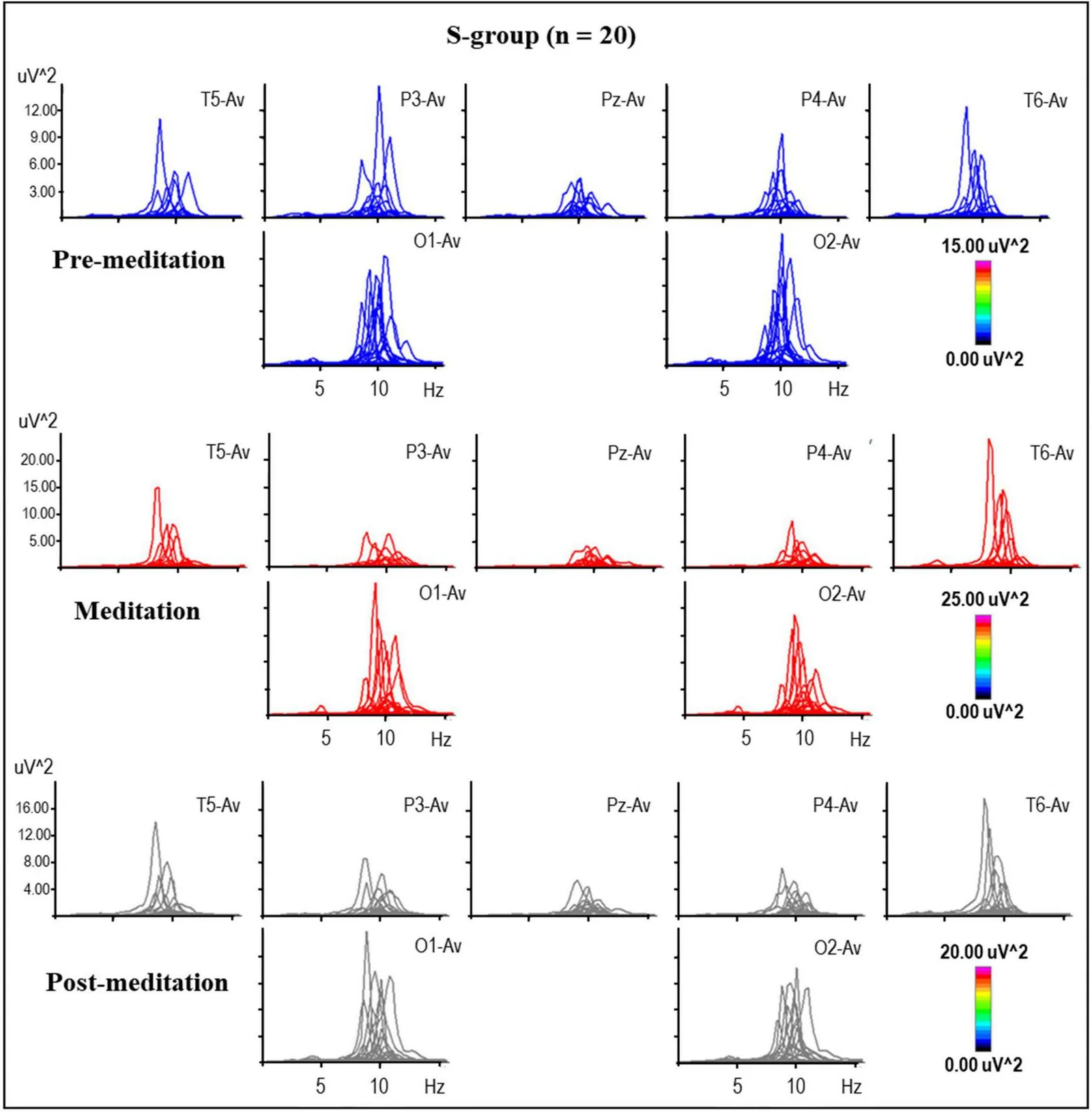
Individual EEG power spectra of each practitioner from S-group, for electrodes T5, P3, Pz, P4, T6, O1 and O2 in the pre-meditation resting baseline state (blue), during meditation (red) and in the post-meditation resting baseline state (gray).

**FIGURE 3:**
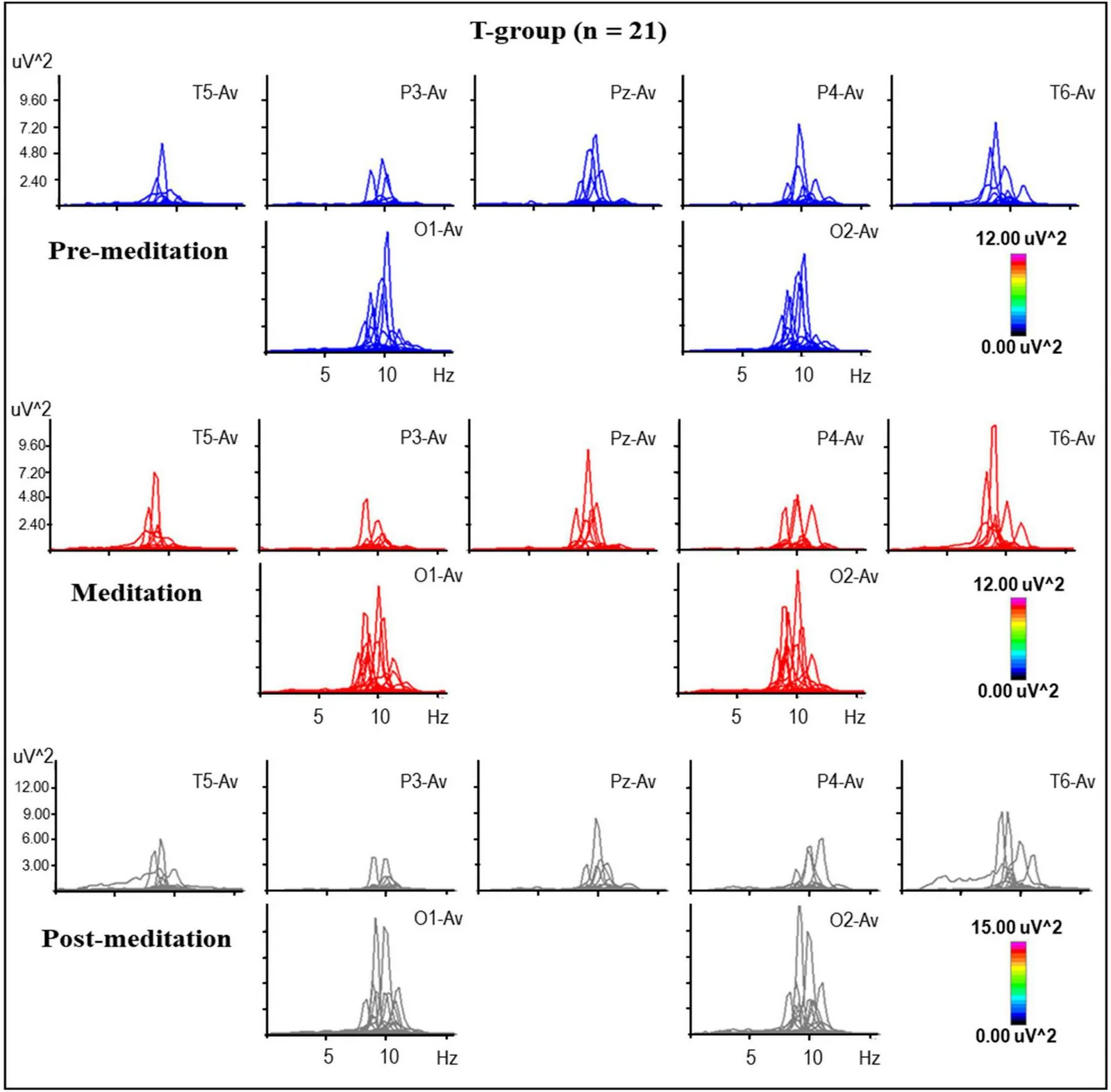
Individual EEG power spectra of each practitioner from T-group, for electrodes T5, P3, Pz, P4, T6, O1 and O2 in the pre-meditation resting baseline state (blue), during meditation (red) and in the post-meditation resting baseline state (gray).

**FIGURE 4:**
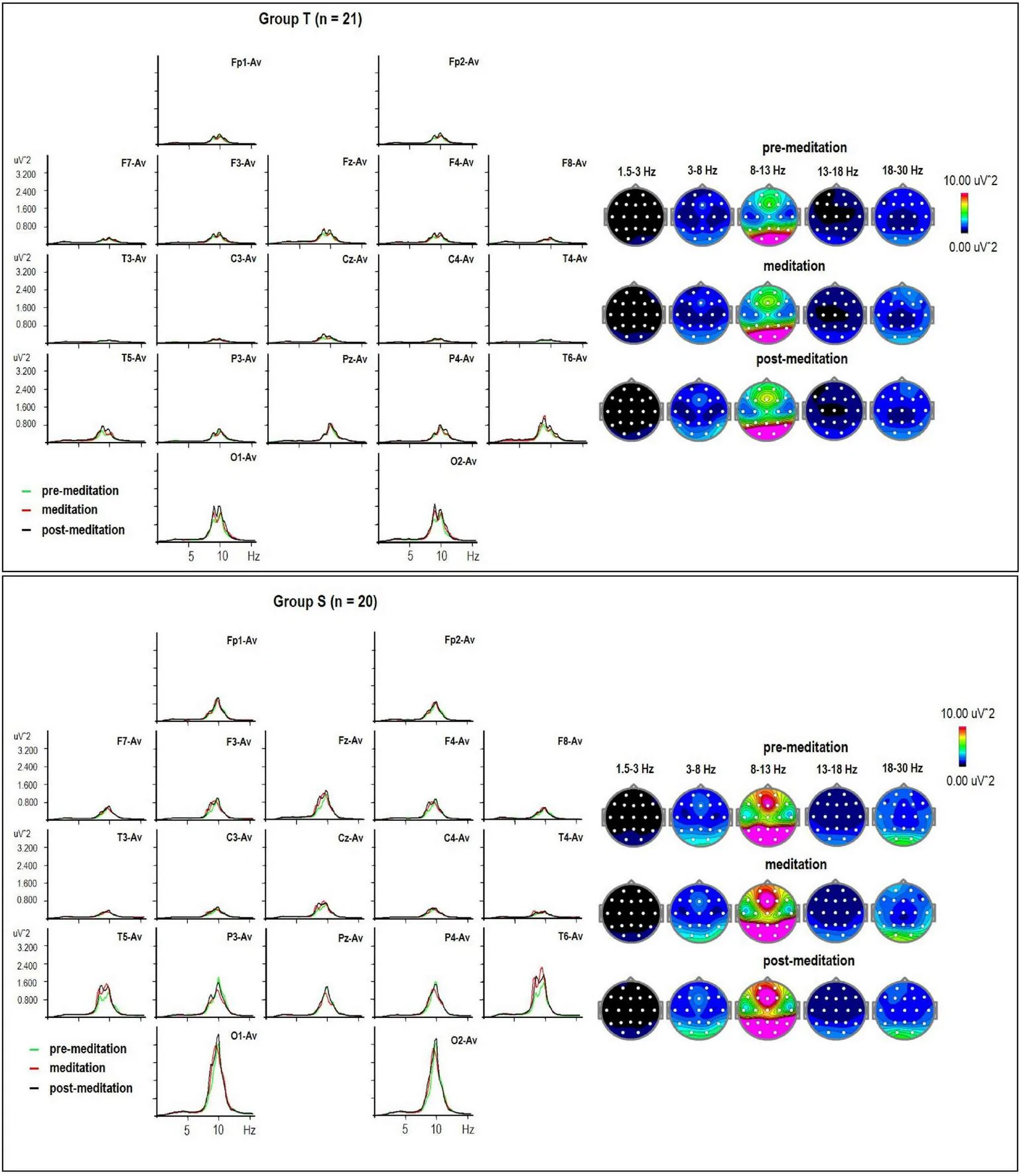
Group-averaged EEG power spectra at pre-meditation resting baseline state (blue), during meditation (red), post-meditation resting baseline state (green).

### 3.3 Individual alpha peak frequency

#### 3.3.1 Within-group analysis

Statistical analysis demonstrated that in the T-group, the iAPF significantly differed across the three conditions (Friedman ANOVA, *p* = 0.00038). The mean values and standard deviations for the T-group were as follows: 9.8 ± 1 Hz before meditation, 9.99 ± 1.13 Hz during meditation, and 9.7 ± 1 Hz after meditation. A pairwise comparison of conditions within T-group (Wilcoxon matched-pairs test) showed a significant increase (*p* = 0.039, *d* = 0.14) in the peak frequency of the alpha rhythm during meditation compared to the pre-meditation resting baseline state and a decrease (*p* = 0.022, *d* = 0.2) in the peak frequency after meditation (Figure 5A).

**FIGURE 5:**
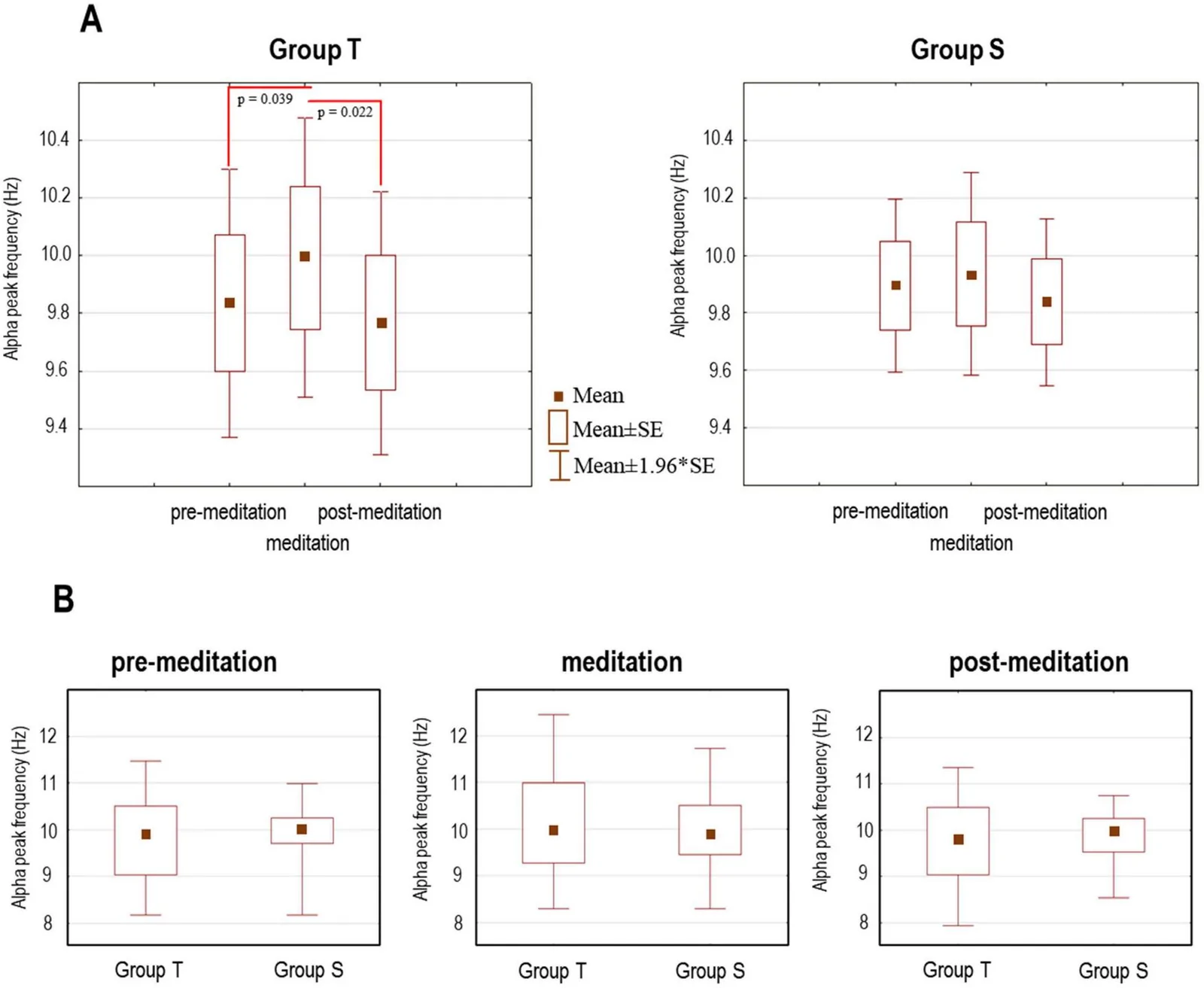
**(A)** The within-group comparison of individual alpha peak frequency (iAPF) for each condition (pre- and post-meditation, meditation) in group T and in group S. **(B)** The between-group comparisons of iAPF in each condition (pre- and post-meditation, meditation). Mean values of iAPF and standard errors of the mean are presented.

In contrast, no significant differences in the peak frequency of the alpha rhythm were observed across conditions in the S-group (Friedman ANOVA, *p* = 0.36). The mean values for the peak frequency in the S-group remained stable: 9.9 ± 0.68 Hz before meditation, 9.9 ± 0.83 Hz during meditation, and 9.9 ± 0.72 Hz after meditation (Figure 5A).

#### 3.3.2 Between-group analysis

Between-group comparison (Mann-Whitney U Test) of iAPF in each condition (before, after and during meditation) did not reveal any significant differences between the groups (Figure 5B).

### 3.4 Additional analysis

Given the differences observed between the groups, an additional comparative analysis of individual subjects’ data was conducted (Table 1 Study population) to identify potential confounding factors.

An additional Mann-Whitney U test revealed no significant between-group differences in age (years) (*p* = 0.20), meditation duration (*p* = 0.92), or subjective success of the practice (rated as 1 - worse than usual, 2 - as usual, 3 - better than usual) (*p* = 0.56). Furthermore, no significant differences were found in discomfort levels (*p* = 0.19) or medication usage (*p* = 0.73), both evaluated on a binary scale (1 - absence, 2 - presence).

Given that between-group differences were observed in alpha power, which is known to fluctuate due to diurnal rhythms and fatigue, we paid special attention to both time of day at study onset and subjective drowsiness. A comparison of the groups regarding subjective drowsiness or fatigue (1 - absence, 2 - presence) revealed that although participants in the S-group reported these symptoms slightly more frequently, the difference was not statistically significant (*p* = 0.19). Regarding the timing of the study, we adhered to the monasteries daily routine by conducting two research sessions per day taking into account the participants’ preferences for their more comfortable practice time. Specifically, the T-group comprised 14 subjects in the morning, and 7 in the afternoon. The S-group consisted of 13 participants in the morning and 7 in the afternoon. Consequently, both groups were highly comparable in terms of experimental timing.

A comparison of the groups in terms of regular practice duration (years) revealed near-significant differences (*p* = 0.054), with T-group participants having a slightly longer history of regular meditation. The groups also differed significantly regarding retreat experience (*p* = 0.025), with the T-group demonstrating a higher prevalence of and longer durations for such practices.

## 4 Discussion

The primary objective of this study was to identify and compare the neurophysiological characteristics of Buddhist meditative practices within the Sutra and Tantra traditions. We investigated changes in EEG spectral power and iAPF across conditions (pre-meditation, meditation, and post-meditation) to identify the specific meditative states characteristic of the Sutra and Tantra traditions. We also conducted a comparative analysis of EEG spectral power and iAPF between two groups of experienced practitioners. We hypothesized that between-groups neurophysiological differences would elucidate the long-term changes in brain function arising from long-term experience in different traditions.

A between-group cluster permutation test demonstrated that the groups of practitioners differ significantly only in EEG alpha power, both during meditation and in the resting baseline states before and after the meditation session. Specifically, the T-group exhibited consistently lower alpha power compared to the S-group. The data in Table 2 shows that the alpha power values in the T-group were approximately half of those in the S-group.

Within-group analysis demonstrated that the conditions (pre-meditation baseline, meditation, and post-meditation baseline) differed only in the T-group and only in iAPF values: during meditation, an increase in iAPF was observed in the occipital leads (O1 and O2), followed by a decrease in iAPF after meditation.

The observed between-group differences in EEG alpha power appear to be independent of whether the subjects are engaged in meditation or remain in a baseline resting state. Thus, the observed differences are stable characteristics of the study groups occurring independently of the conditions.

What could explain these alpha differences between the groups?

In the literature, different meditation practices are associated with changes in virtually all EEG frequency bands, from delta to gamma (see reviews: Cahn and Polich, 2006; Fell et al., 2010; Travis and Shear, 2010; Brandmeyer et al., 2019; Fox and Cahn, 2021). Therefore, we also expected to find more extensive differences between the two groups. However, the fact that significant differences were identified specifically within the alpha frequency band is well-supported by the literature. The alpha rhythm has long been regarded as a key neurophysiological marker of attentional processes and mental states in various meditation traditions. Increased alpha activity has frequently been observed during meditation compared to control conditions, regardless of the specific technique or the practitioners’ level of expertise. Furthermore, heightened alpha activity has been identified in various groups of meditators when compared to control subjects. Several studies have also reported shifts - primarily a slowing - of the alpha peak frequency during meditation (see reviews: Cahn and Polich, 2006; Fell et al., 2010; Travis and Shear, 2010; Brandmeyer et al., 2019; Fox and Cahn, 2021). Taken together, these findings suggest that alpha activity is not only associated with the meditative states but may also reflect neurophysiological alterations resulting from long-term meditative experience (meditative traits). This independence from the specific type of meditation has been interpreted by some authors as an indication that the alpha rhythm reflects general accompanying processes–such as relaxation or increased mental attention–rather than mechanisms unique to the meditation itself (Fell et al., 2010).

Historically, alpha activity was viewed as a marker of relaxed wakefulness or a neural “idling” state (Pfurtscheller et al., 1996). However, a growing body of experimental evidence suggests that modulations in the alpha band represent active cognitive processes. Current models propose that alpha power dynamics serve as an active mechanism for top-down attentional modulation and the regulation of cortical arousal (Klimesch et al., 2007). Specifically, increased alpha activity is associated with the functional inhibition of cortical areas that are irrelevant to the current task. For instance, during periods of heightened interoceptive attention, an increase in alpha activity plays a crucial role in shielding the brain from external sensory stimuli during the execution of mental tasks (Ray and Cole, 1985; Cooper et al., 2003). Increased alpha power has also been linked to the maintenance of tonic alertness, whereas a decrease in alpha activity is typically observed during selective attention (Sadaghiani et al., 2010). It is hypothesized that while both tonic alertness and selective attention enhance overall attentional states, they employ antagonistic mechanisms. Due to its non-selective nature, tonic alertness involves top-down inhibition (marked by increased alpha power), whereas selective attention involves specific, focal disinhibition or the release of this suppression (marked by decreased alpha power) (Klimesch et al., 2007).

Investigated meditations in the Sutra tradition include analytical meditation and FAM, during which practitioners perform an analytical inquiry into core Buddhist philosophical concepts, penetrating their essential meaning, and/or focusing their entire attention on a given object (Wallace, 2009; Dalai Lama et al., 2019). These practices are associated with maintaining an optimal level of arousal, when a stable balance between hyperarousal and drowsiness is maintained over long periods of time (Thubten, 1999, Britton et al., 2013). These types of meditation have also been shown previously to be more associated with tonic alertness (Amihai and Kozhevnikov, 2014). Therefore, during these practices, one can expect an increase in alpha power as an indicator of active top-down inhibitory processes in the cerebral cortex. This process, during these meditations, may serve to suppress the processing of irrelevant (external) signals, thereby facilitating focusing and sustaining attention on a single mental object, a key requirement for these meditations.

The investigated meditations in the Tantra tradition are also rely on the foundation of one-pointed concentration. However, they also engage cognitive processes such as maintaining complex visualizations of deities and internal bodily processes, retrieving information from memory, and maintaining certain emotional states (Tsongkhapa et al., 2013; Blo-Bzacn-Byams and Engle, 2019; Kittay and Lobsang, 2020). Engaging such a broad range of cognitive processes likely requires activation of various brain regions. Alternatively, performing these practices may require increased selective attention. In either case, it can be hypothesized that a decrease in alpha rhythm power would be observed during these practices, indicating focal disinhibition of the cerebral cortex.

Assuming that regular meditation induces neuroplastic remodeling of brain function, and that temporary changes (meditative states) can develop into long-term changes in brain activity (meditative traits) with experience, then it is possible that the observed between-group alpha differences reflect the result of this process.

Since this study was conducted within a single socio-cultural context, inside monasteries that safeguard the tradition and ensure a uniform format and quality of meditative practices, and because the groups of practitioners are comparable in parameters such as education, lifestyle, and religious beliefs, these parameters could not be the cause of the observed between-group differences. Additional analysis revealed that the groups did not differ in age, meditation duration, subjective success, discomfort during the study, or medication use. Given that between-group differences were observed in alpha power, which can fluctuate due to diurnal rhythms or fatigue, it is important to emphasize that the groups did not significantly differ in subjective drowsiness. The groups were also comparable regarding the time of day the sessions were conducted. Marginally significant between-group differences were found only for regular practice duration, with T-group participants having a slightly longer history of meditation. The only significant between-group differences were related to retreat duration with the T-group demonstrating more extensive retreat experience. This latter fact does not directly explain the lower alpha power values found in the T-group, since most studies associate the presence and depth of meditative experience with an increase in alpha power (Cahn and Polich, 2006; Cahn et al., 2010; Fell et al., 2010). At the same time, existing literature has largely unexamined tantric meditative practices specifically, thus the observed decrease in alpha power may reflect unique, experience-dependent traits specific to tantric tradition.

Futhermore, we cannot entirely rule out the possibility that unmeasured phenomenological or confounding factors may have influenced the observed between-group differences. For instance, the groups might have exhibited baseline differences in psychological profiles, such as distinct cognitive styles or personality traits. At this stage of the research, we were unable to conduct a psychological assessment of the participants, as this would have required the cross-cultural validation and psychometric adaptation of existing questionnaires to account for the unique linguistic, socio-cultural, and religious characteristics of the monastic community. Developing and validating such culturally adapted tools is an important and promising direction for further research on meditative practices in Buddhist monasteries.

While EEG alpha activity has been extensively studied, several questions remain open regarding its neural sources and functional significance (Hughes and Crunelli, 2007). Furthermore, some perspectives suggest the existence of multiple alpha sources (Başar et al., 1997; Schürmann and Başar, 2001; Ben-Simon et al., 2008), implying that the human alpha rhythm reflects several concurrent processes. Therefore, further research with larger sample sizes is required to elucidate the mechanisms underlying these band-specific intergroup differences, their functional significance, and the absence of variations in other EEG frequency bands.

Except for the iAPF dynamics in the T-group, the lack of significant spectral variations across conditions accords with several previous neurophysiological studies. Several previous studies have also demonstrated that comparing meditation with a baseline resting condition may not always yield the expected pronounced changes in EEG parameters. For instance, similar results were reported in research on Transcendental Meditation (TM) (Tebecis, 1975), where meditative states showed little contrast with rest, yet significant meditative traits were identified when comparing the practitioner group with a non-meditating control group during the resting condition. Furthermore, it has been demonstrated that meditation training (e.g., an 8-weeks course in Taoist meditation) leads to significant changes in EEG activity during resting states when comparing pre- and post-training baselines. However, no significant alterations in EEG parameters were observed during the actual meditation process when comparing the beginning and the end of the training period (Rusinova et al., 2024).

On one hand, it is often hypothesized that experienced practitioners should demonstrate more pronounced changes in brain activity when comparing meditative and non-meditative conditions. On the other hand, if the ultimate goal of meditation is to transform the baseline state, then the neurophysiological differences between formal meditation and baseline resting condition should diminish with increased expertise. For example, Travis et al. (2002) noted that a number of long-term TM practitioners reported that transcendental experiences, which initially emerged only during their formal practice, now subjectively coexist with their waking and sleeping states outside of meditation. Indeed, long-term practice is expected to reduce the gap between formal meditation and daily life, leading to a convergence of meditative state and trait. In this regard, for highly experienced practitioners - such as those in our study - we may indeed observe no pronounced changes in brain activity parameters when comparing states outside of meditation with the meditative state itself.

However, in our case, analysis of individual EEG power spectra revealed that individual subjects exhibited changes in spectral power between pre-meditation, during meditation, and post-meditation conditions, but these differences varied significantly across participants. Figures 2, 3 show the variability of individual EEG power spectra under three conditions (pre-meditation, during meditation, and post-meditation). It is likely this diversity of individual patterns that led to the lack of statistically significant changes at the group level.

It can be assumed that these individual differences within the groups arise because highly experienced practitioners, over years of practice, develop their own subject-specific techniques and strategies for maintaining the meditative state. As expertise deepens, meditation may acquire increasingly idiosyncratic characteristics, reflected in diverse patterns of brain activity. Such pronounced inter-individual variability can mask statistically significant differences between states when data are averaged at the group level. For instance, a recent study (Volodina et al., 2021) involving both experienced practitioners and novices demonstrated that during a guided Taoist meditation, the expert group bifurcated into two distinct subgroups based on the nature of their physiological changes. This led the authors to hypothesize that even within a single meditative technique, experienced practitioners may exhibit divergent physiological correlates. These findings further support the notion that long-term expertise often results in individualized physiological patterns of meditation. In more general terms, mastery may not unify physiology, but, on the contrary, can make it more individual and segmented.

At the same time, we cannot exclude that some additional factors could have influenced the individual variability of the data. For example, within the S-group, 12 participants performed analytical meditation, four performed FAM, and four engaged in a mixed meditation type (where the participant periodically switches to analytical processes to restore focus during FAM). At this stage of the research, the small sample size precluded us from distinguishing between these specific meditation types within the Sutra tradition. However, it has previously been shown that these two types of meditation can be accompanied by different EEG correlates (Neri et al., 2024). In the T-group, 10 subjects performed the Guhyasamāja practice, 11 subjects performed the Yamāntaka practice. Although at this stage of the study, based on textual descriptions and verbal explanations, we assumed that the differences between these practices which belong to one category (Tantra) are minor, this point should nevertheless be verified in the future. The lack of significant changes in EEG spectral power between conditions may also be linked to the individual variables presented in Table 1. However, the limited sample size in this study precludes us from examining how these variables and their interactions influence the observed EEG variations. Interindividual variability may also be linked to unmeasured phenomenological factors. Our further research will focus on quantitative analysis of this variability on a larger sample of subjects.

The only difference found between the conditions was an increase in iAPF values during meditation, followed by a decrease after meditation in the T-group. Although the observed changes in individual alpha peak frequency were not substantial, the fact that iAPF dynamics were detected only during tantric meditations characterizes them uniquely. Given that literature on meditation-induced iAPF changes is largely underreported, these results offer valuable insights into the neurophysiological mechanisms organizing brain activity during contemplative practices. Furthermore, while prior studies predominantly report decreases in iAPF values or a general slowing of the alpha rhythm during meditation (Zhang et al., 1988; Saggar et al., 2012), our findings demonstrate a contrary trend, characterized by a significant increase in iAPF during Tantra practices.

On the one hand, iAPF is a stable individual neurophysiological marker; on the other hand, iAPF values can change depending on a person’s functional state. For example, the individual alpha peak frequency has been shown to increase in response to higher cognitive demands and greater task engagement (Angelakis et al., 2004; Haegens et al., 2014). It has been hypothesized that the alpha peak frequency reflects the temporal resolution of brain function: a higher frequency facilitates a more rapid alternation between excitatory and inhibitory phases, thereby increasing the information processing speed (Klimesch et al., 1993). Thus, it can be assumed that the increase in iAPF during tantric meditations is due to the fact that these meditation techniques are associated with the manipulation of complex dynamic mental images and this requires an increase in the speed of mental information processing in the brain.

## 5 Challenges and limitations

A primary limitation of this study is the absence of a meditation-naïve control group and the lack of longitudinal data. This constraint, to some extent, narrows the interpretation of the observed between-group differences as true “meditative traits.” The absence of a control cohort in this study is largely due to the substantial logistical challenges in recruiting a meditation-naïve group that is demographically and culturally matched to a population of practicing Buddhist monks of Tibetan ethnicity. For example, non-meditating laypeople from Tibetan settlements around monasteries cannot be used as a control group, as the lifestyles of monks and laypeople differ significantly. Recruiting a control group of non-meditating subjects within the monastic community is also impossible, as these would be young monks who have not yet begun regular meditation practice, and in this case, the study groups would differ in age. Therefore, in this study, we hypothesized that long-term experience in different meditation traditions may also lead to long-term changes in brain activity, and these changes may be detectable to some extent when comparing groups of subjects from different traditions. Furthermore, since Tantric meditation relies heavily on the skills developed through Sutra practices, we suggested that the S-group could, to some extent, be seen as a control group for Tantric practitioners.

Since this study revealed certain between-group differences, the comparability of the participants groups should be examined in more detail. A key characteristic of this study is that it was conducted within a uniform socio-cultural and religious framework, specifically inside monasteries that serve as guardians of the tradition and ensure a standardized format and high caliber of meditative practices. Consequently, the examined cohorts are highly comparable across key sociodemographic and lifestyle parameters, including education, daily routines, and religious beliefs. Additional analysis showed that the groups were matched on most parameters presented in Table 1 with the only significant differences pertaining to the experience factor. While these between-group variations may indeed be driven by experience, the influence of other unmeasured factors cannot be entirely ruled out. For instance, a notable limitation of this study is the absence of psychometric profiling, such as assessments of cognitive styles or standardized personality questionnaires. It cannot be ruled out that psychological assessment would have further clarified the underlying mechanisms behind the identified between-group differences. The absence of a psychological component is due to the rigorous validation required to adapt existing metrics to this specific socio-cultural environment. Undertaking this psychometric validation is a highly valuable pursuit for future research.

Further limitations include the cross-sectional design, a relatively small sample size, the absence of precise behavioral or cognitive outcome assessments, and the exclusively male composition of the groups, which precludes causal inferences. Future studies should expand the cohort to include Buddhist nuns, providing a more diverse demographic framework. Given that all participants were experienced male Tibetan Buddhist monks, the generalizability of these findings to other demographic groups may be limited.

Our results revealed significant variation in individual subjects’ responses to the conditions before, after, and during meditation. This inter-individual variability was observed in both groups of subjects and was independent of the Sutra or Tantra tradition. Therefore, future research should focus on a more detailed quantitative analysis of individual variability within study groups. In particular, Table 1 presents details on the study population, demonstrating that certain gradations of personal characteristics can be identified within the groups. However, the limited sample size precludes a detailed analysis of how these variables and their interactions influence the observed between-condition EEG differences.

In recent years, machine learning and deep learning methods have been increasingly applied to the study of traditional contemplative practices (Das and Deb, 2026; Ohri and Dutta, 2026). Research demonstrates that these algorithms can effectively distinguish between diverse brain states, including those during meditation (Singh et al., 2025; Ghadiyaram et al., 2025; Das et al., 2026). Furthermore, integrating EEG with other neuroimaging or physiological modalities represents a powerful paradigm for deepening our understanding of the brain-body interactions underlying meditative techniques (Das and Deb, 2026). Given that our research typically employs a multimodal approach–incorporating parallel recordings of EEG, electrocardiogram - ECG and infrared thermography–combining these data streams with enriched phenomenological insights and machine learning algorithms in future studies will help elucidate the identified trends and address the questions raised in this article.

## 6 Summary and conclusion

Between-group analysis revealed that the Tantra and Sutra cohorts exhibited significant differences in EEG alpha power, which persisted during meditation as well as across both pre- and post-meditation resting baselines. Tantra practitioners demonstrated consistently lower alpha power compared to the Sutra group, regardless of the conditions. Thus, the observed differences are stable characteristics of the examined groups. We suggest that reported between-group differences represent enduring meditative traits developed through years of training in meditative techniques in the Sutra and Tantra traditions. Given that the groups differ in both the prevalence and duration of retreats, the observed between-group differences may be also driven by this variation in retreat experience. Nevertheless, we cannot entirely exclude the possibility that these variations may also stem from certain cohort-specific characteristics unrelated to meditation.

The within-subjects analysis demonstrated that the conditions (meditation, and baseline resting conditions before and after meditation) differed only in the Tantra group: during meditation, an increase in individual alpha peak frequency (iAPF) was observed in the occipital leads, followed by a decrease in iAPF after meditation. An examination of individual power spectra revealed that the lack of significant differences in EEG spectral power between conditions is likely due to pronounced inter-individual variability in both groups. It can be assumed that this inter-individual variability may be attributed to the fact that highly experienced practitioners, over years of training, develop highly individualized cognitive strategies and idiosyncratic techniques for maintaining the meditative state. Our future research will focus on a more detailed quantitative analysis of this inter-individual variability during meditations and on identifying additional between-group differences among practitioners in different traditions.

## Data Availability

The raw data supporting the conclusions of this article will be made available by the authors, without undue reservation.

## In memoriam

We also express the deepest gratitude and honor the memory of the late Professor Svyatoslav Medvedev, the project’s principal investigator and organizer.
